# Generalist hydrocarbon-degrading bacterial communities in the oil-polluted water column of the North Sea

**DOI:** 10.1111/1751-7915.12176

**Published:** 2014-09-24

**Authors:** Panagiota-Myrsini Chronopoulou, Gbemisola O Sanni, Daniel I Silas-Olu, Jan Roelof van der Meer, Kenneth N Timmis, Corina P D Brussaard, Terry J McGenity

**Affiliations:** 1 School of Biological Sciences, University of EssexWivenhoe Park, Colchester, CO4 3SQ, UK; 2 Department of Fundamental Microbiology, University of LausanneLausanne, Switzerland; 3 Institute of Microbiology, Technical University BraunschweigBraunschweig, Germany; 4 Department of Biological Oceanography, Royal Netherlands Institute for Sea Research (NIOZ)Den Burg, The Netherlands

## Abstract

The aim of this work was to determine the effect of light crude oil on bacterial communities during an experimental oil spill in the North Sea and in mesocosms (simulating a heavy, enclosed oil spill), and to isolate and characterize hydrocarbon-degrading bacteria from the water column. No oil-induced changes in bacterial community (3 m below the sea surface) were observed 32 h after the experimental spill at sea. In contrast, there was a decrease in the dominant SAR11 phylotype and an increase in *Pseudoalteromonas* spp. in the oiled mesocosms (investigated by 16S rRNA gene analysis using denaturing gradient gel electrophoresis), as a consequence of the longer incubation, closer proximity of the samples to oil, and the lack of replenishment with seawater. A total of 216 strains were isolated from hydrocarbon enrichment cultures, predominantly belonging to the genus *Pseudoaltero monas*; most strains grew on PAHs, branched and straight-chain alkanes, as well as many other carbon sources. No obligate hydrocarbonoclastic bacteria were isolated or detected, highlighting the potential importance of cosmopolitan marine generalists like *Pseudoalteromonas* spp. in degrading hydrocarbons in the water column beneath an oil slick, and revealing the susceptibility to oil pollution of SAR11, the most abundant bacterial clade in the surface ocean.

## Introduction

The global use of crude oil has plagued the marine environment with numerous major oil-pollution incidents, resulting in devastating environmental damage to marine habitats with serious socio-economic implications. A recent example is the largest offshore spill in the history of the USA, which occurred when the Deepwater Horizon rig exploded, releasing several hundred million litres of oil into the Gulf of Mexico (Crone and Tolstoy, 2010). Crude-oil components are toxic and stressful to marine organisms, including microorganisms (Sikkema *et al*., 1995), and if they reach the shore they can persist for decades (Atlas and Bragg, 2009). However, many microbes have developed pathways for hydrocarbon metabolism, some to the extent that they thrive only in the presence of crude-oil components (Dyksterhouse *et al*., 1995; Yakimov *et al*., 1998; 2004a; Teramoto *et al*., 2011; McGenity, 2014). Following oil contamination in marine systems, these obligate hydrocarbonoclastic bacteria (such as *Alcanivorax*, *Cycloclasticus*, *Thalassolituus*, and *Oleibacter* species) typically bloom and become dominant members of the prevailing microbial communities (Kasai *et al*., 2002; Cappello *et al*., 2007; McKew *et al*., 2007b; Teramoto *et al*., 2009; Vila *et al*., 2010).

These specialized bacteria are often found in close contact with oil at the oil-water / oil-sediment interface (Schneiker *et al*., 2006; Gertler *et al*., 2009; Cappello and Yakimov, 2010). In experimental oil-contaminated tidal mudflat mesocosms, Coulon and colleagues (2012) showed that *Alcanivorax* sp. constituted almost 50% of the community in the tidal biofilms floating above oiled mudflat cores. *Thalassolituus* sp. dominated the microbial communities present in the oily phase of water samples obtained from production wells in Canada (Kryachko *et al*., 2012). And, in beach-simulating mesocosms, *Alcanivorax* and *Cycloclasticus* dominated the surfaces of seawater-immersed oil-coated gravel after the addition of nutrients (Kasai *et al*., 2002). Microbes like *Alcanivorax borkumensis* are exquisitely adapted to biofilm formation, oil solubilization and degradation, and oil-induced stress tolerance (Schneiker *et al*., 2006; Sabirova *et al*., 2008).

However, oil floating on the surface of marine waters is broken down into small droplets, dispersed and integrated into the water column to different degrees depending on many factors including wave action (ITOPF, 2002; Gros *et al*., 2014). Known obligate hydrocarbonoclastic bacteria are not universally encountered in the water column below an oil slick, for example in the Gulf of Mexico (Redmond and Valentine, 2011). Therefore, we hypothesize that below the oil-water interface, specialized hydrocarbonoclastic bacteria are replaced by generalist marine bacteria, perhaps better adapted to grow on low levels or more soluble components of crude oil. Besides, there is paucity of information on the effect of crude oil spills on marine bacteria that play vital roles in global biogeochemical cycles. Therefore, we sought to investigate the impact of an oil spill on pelagic marine bacteria, both *in situ* and in mesocosms, that were not in direct contact with the oil slick; and to test the hypothesis that some common marine bacteria would be inhibited by the oil. Consequently, we have carried out comparative studies of bacterial community changes in the water column during a small experimental spill of light crude oil in the North Sea and in oil-enriched 1-m^3^ seawater mesocosms. Furthermore, we have isolated, identified and characterized generalist hydrocarbon-degrading microorganisms from both systems.

## Results

### Bacterial community changes during the experimental oil spill and in the oil-enriched on-board mesocosms

**Fig 1:**
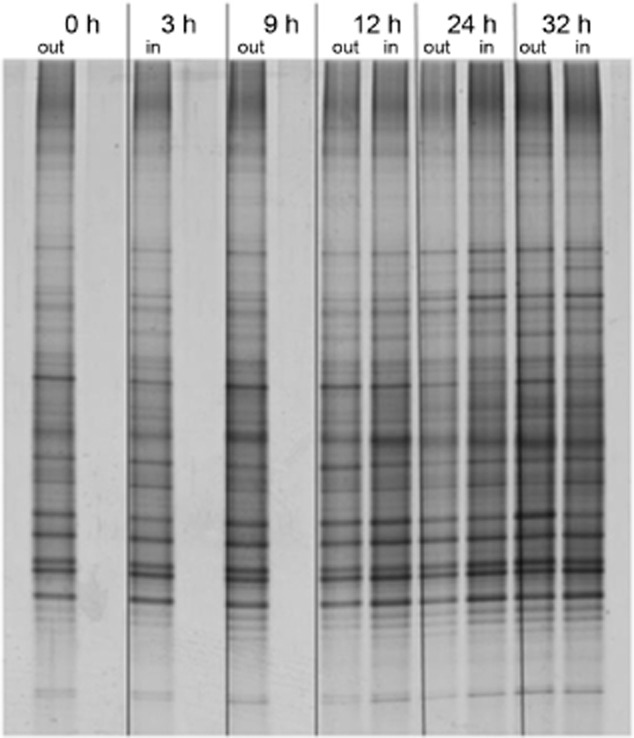
DGGE profiles of amplified bacterial 16S rRNA genes from 1.5 m (out) and 3 m (in) below the sea surface during the experimental oil spill (outside and inside the spill respectively) at different times. Refer to the *Experimental Procedures* for an explanation of the experimental-spill and sampling strategy.

**Fig 2:**
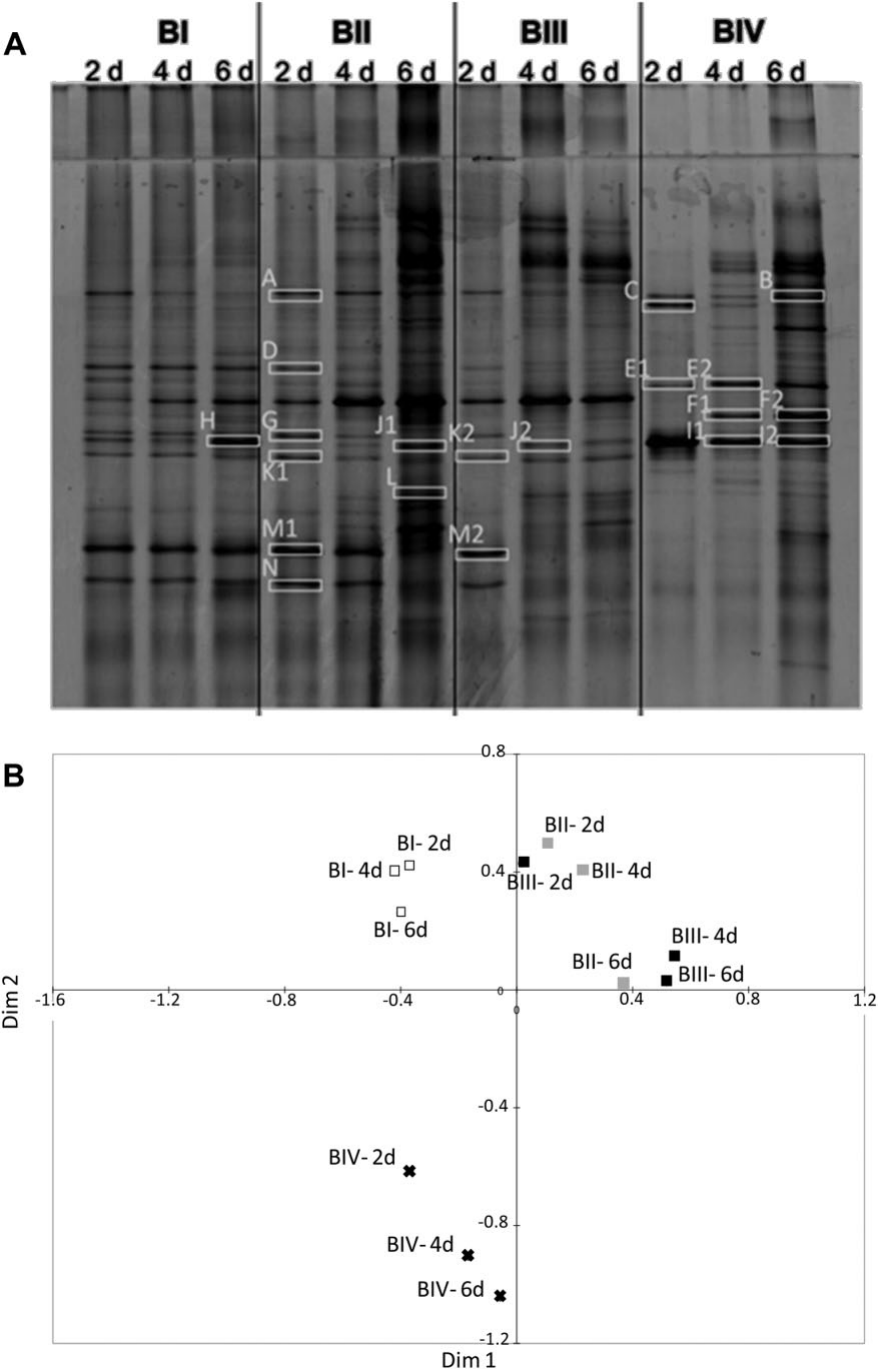
DGGE profiles of amplified bacterial 16S rRNA genes from the on-board mesocosms (days 2, 4 and 6).A. Profiles of days 2, 4 and 6 of all the four mesocosms are shown. The bands in white rectangles were excised and sequenced. BI: no oil (control); BII: 2 L of Arabian Light crude oil; BIII: 5.5 L of Arabian Light crude oil; BIV: 2 L of Arabian Light crude oil and UV treated (killed control).B. MDS plot of the DGGE profiles of the on-board mesocosms. White squares indicate the non-oiled mesocosm (BI), grey squares the less oiled (BII), black squares the most oiled (BIII) and UV treated mesocosm is indicated by an ‘x’.

Temporal and treatment effects on the bacterial communities in the experimental oil spill at sea and the oil-enriched enclosed mesocosms on-board the ship were investigated by denaturing gradient gel electrophoresis (DGGE) analysis of the bacterial 16S rRNA gene. There was no obvious difference between water-column bacterial communities inside and outside of the experimental light crude oil spill at sea over 32 h (Fig. 1). In the on-board mesocosms, where samples could be taken closer to the surface (∼ 15 cm), and the experiment was performed over a longer period, clear differences over time and between treatments were seen (Supplementary Fig. S1 and Fig. 2A). The community of the non-oiled mesocosm (BI) was relatively stable over the course of the experiment. In contrast, there were changes in the oiled mesocosms (BII and BIII) from day 2 until day 4, at which time the communities became more stable (e.g. compare BI and BIII in Supplementary Fig. S1). The multidimensional scaling (MDS) plot (Fig. 2B) shows that addition of oil had a major impact on the bacterial community composition; the profiles of the two oiled mesocosms (BII, BIII) are distinct from the non-oiled mesocosm (BI), yet are relatively similar at corresponding time points, except that changes occur earlier in the heavily oiled mesocosm (BIII). The bacterial community surviving and then growing in the UV-treated mesocosm (BIV) was the most different, becoming more species rich over time (Fig. 2), showing that UV treatment did not kill all microbes, but selected for a distinct bacterial community.

Bacterial identification by sequencing DGGE bands was done for the on-board mesocosms, focusing on those bands that were most influenced by oil addition (Fig. 2A, Table 1). The main bacteria detected belong to the alphaproteobacterial SAR11 group and the gammaproteobacterial genus *Pseudoalteromonas*. Bands similar to SAR11 types (97–100% 16S rRNA gene identity) were present in the non-oiled mesocosm (BI), in the less oiled mesocosm (BII) and at early time points of the most oiled mesocosm (BIII). However, when oil was present at high concentration or lower concentration but for longer, many bands (D, M1, M2, N) representing SAR11 bacteria, were absent. Nevertheless, some SAR11*-*related bands persisted in oiled mesocosms, namely A, K1, K2. In contrast, the bands from *Pseudoalteromonas* spp. increased in intensity in oiled mesocosms, for example band J1, appearing at day 6 in the less-oiled mesocosm, and band J2 present at day 4 and 6 in the most oiled mesocosm (Fig. 2A). Band L, which was present at day 6 in the less-oiled mesocosm, was also similar (98% 16S rRNA gene sequence identity) to *Pseudoalteromonas* sp. Although the UV-treated and oiled mesocosm (BIV) is a very artificial system, having selectively killed certain microbes, the dominant bands over time were most similar to *Pseudoalteromonas haloplanktis*. For example, bands I1 (99%) and I2 (99%), present at all time points were both similar to this species. Band E1 was also similar (99%) to *Pseudoalteromonas* sp. In this mesocosm there were also some dense bands, such as band B (97%) and bands F1 (99%) and F2 (100%), similar to *Alteromonas* spp.

**Table 1:** Sequencing results of the 16S rRNA gene DGGE bands from the on-board mesocosms

Band	Oil Band int.^a^	Mesocosms -Time^b^	Closest Relative	Group^c^	Accession No	Id (%)^d^
A	—	BII-2 day	SAR11 bacterium	Alpha	JQ859613	99
B	↑	BIV-6 day	*Alteromonas macleodii*	Gamma	CP003873	97
C	↓	BIV-2 day	*Pseudoalteromonas* sp. M71_D76	Gamma	FM992730	99
D	↓	BII-2 day	SAR11 bacterium	Alpha	HM799000	99
E1	↑	BIV-2 day	*Pseudoalteromonas* sp. MSI02	Gamma	EU420037	99
E2	↑	BIV-4 day	*Pseudomonas* sp. 28ox	Gamma	JQ411283	99
F1	↑	BIV-4 day	*Alteromonas* sp. B348	Gamma	FN295800	99
F2	↑	BIV-6 day	*Alteromonas tagae* BCR 17571	Gamma	NR_043977	100
G	↓	BII-2 day	SAR11 bacterium	Alpha	JN710109	100
H	↓	BI-6 day	Uncultured marine bacterium	Beta	AF419359	99
I1	↑	BIV-4 day	*Pseudoalteromonas* sp. BB68	Gamma	FR693362	99
I2	↑	BIV-6 day	*Pseudoalteromonas* sp. BB68	Gamma	FR693362	99
J1	↑	BII-6 day	*Pseudoalteromonas marina* ECSMB2	Gamma	JX206469	100
J2	↑	BIII-4 day	*Pseudoalteromonas* sp. BSi20652	Gamma	BADT01000269	98
K1	↓	BII-2 day	SAR11 bacterium	Alpha	HM799000	98
K2	—	BIII-2 day	SAR11 bacterium	Alpha	JQ859613	97
L	↑	BII-6 day	*Pseudoalteromonas marina* ECSMB2	Gamma	JX206469	98
M1	↓	BII-2 day	SAR11 bacterium	Alpha	JQ859613	100
M2	↓	BIII-2 day	SAR11 bacterium	Alpha	JQ859613	99
N	↓	BII-2 day	SAR11 bacterium	Alpha	JQ859613	100

Sequences are based on approximately 130 bp of the 16S rRNA gene. A BLAST search was performed against the NCBI database.

a Arrows indicate increase (↑) or decrease (↓) over time in oiled mesocosms; Dash symbol (—) indicates no change in band intensity.

b BI: no oil (control); BII: 2 L of Arabian light crude oil; BIII: 5.5 L of Arabian light crude oil; BIV: 2 L of Arabian light crude oil and UV light-treated (killed control).

c Alpha = Alphaproteobacteria; Beta = Betaproteobacteria; Gamma = Gammaproteobacteria.

d Percentage partial 16S rRNA gene sequence identity.

### Isolation and characterization of oil-degrading strains

Although DGGE analysis suggests that *Pseudoalteromonas* spp. are probably the main microbes responsible for the degradation of oil in the on-board mesocosms, it is known that many species of this genus do not degrade hydrocarbons. Consequently, *Pseudoalteromonas*-related bacteria were cultivated to test the role they play in hydrocarbon degradation.

Samples from the experimental spills (Spill-in), accidental slicks (Acc-in) and their non-contaminated controls (Spill-out, Acc-out) and oil-enrichment mesocosms were inoculated into the hydrocarbon-enrichment media, and after subculturing, 216 strains were isolated (Table 2); 47 from the experimental spills [40 strains from inside the spill (Spill-in) and 7 from outside the spill (Spill-out)], 50 from the accidental slicks [25 each from inside the slicks (Acc-in) and outside (Acc-out)], 119 from the on-board mesocosms.

**Table 2:** Summary of strains isolated from hydrocarbon-enrichments of North Sea samples

		Number of Isolates						
			Carbon Source^d^				Media	
Environment		TOTAL	T	F	P	M	Solid	Liquid
ON-BOARD MESOCOSMS^a^	BI	**25**	6	8	9	2	12	13
	BII	**26**	12	9	3	2	9	17
	BIII	**36**	15	12	6	3	16	20
	BIV	**32**	11	10	2	9	18	14
EXPERIMENTAL SPILL^b^	Spill-in	**40**	13	17	10	0	19	21
	Spill-out	**7**	3	1	3	0	6	1
ACCIDENTAL SLICKS^c^	Acc-in	**25**	9	10	5	1	14	11
	Acc-out	**25**	8	9	6	2	8	17
	**TOTAL**	**216**	77	76	44	19	102	114

a BI: no oil (control); BII: 2 L of Arabian light crude oil; BIII: 5.5 L of Arabian light crude oil; BIV: 2 L of Arabian light crude oil and UV light-treated (killed control).

b Spill-in: inside experimental oil spill; Spill-out: outside the experimental spill.

c Acc-in: inside accidental slicks; Acc-out: outside accidental slicks.

d T: tetradecane; F: weathered Forties crude oil; P: pristane; M: 1-methyl naphthalene.

**Fig 3:**
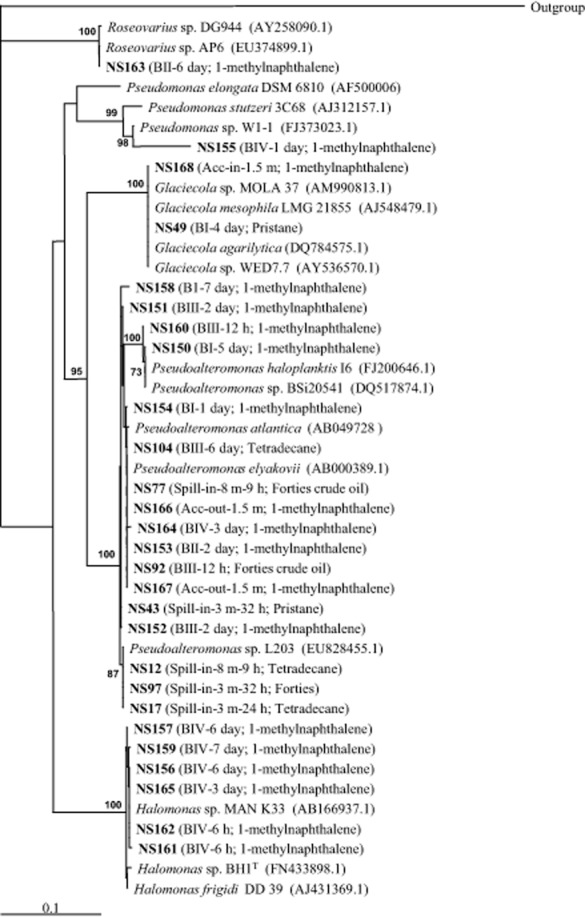
Phylogenetic tree of North Sea isolates based on partial 16S rRNA (500 bp) sequences. Spill-in: inside experimental oil spill; Spill-out: outside experimental spill; Acc-in: inside accidental slicks; Acc-out: outside accidental slicks. On-board mesocosms – BI: no oil (control); BII: 2 L of Arabian Light crude oil; BIII: 5.5 L of Arabian Light crude oil; BIV: 2 L of Arabian Light crude oil and UV treated (killed control). The bar represents the average nucleotide substitution per base. The 16S rRNA gene of *Haloferax volcanii* NCIMB 2012 (AY425724.1) was used as outgroup.

Most strains were isolated from the tetradecane (77 strains) and crude oil (76 strains) enrichment cultures, while 19 strains were isolated from enrichment cultures with 1-methylnaphthalene as the sole carbon source. Sequencing of 16S rRNA genes from all 1-methylnaphthalene-grown isolates and selected isolates from other hydrocarbon-enrichments revealed a predominance of generalist hydrocarbon-degrading members belonging to Gammaproteobacteria but not obligate hydrocarbonoclastic bacteria (Fig. 3). With the exception of strain NS168 related to *Glaciecola* sp. (100% 16S rRNA gene sequence identity), all the isolates from samples obtained from both within and outside the spill and accidental slicks belonged to the genus *Pseudoalteromonas* (99.4–100% identity). Strain NS163 (99% identity to *Roseovarius* strain AP6), isolated from a 1-methylnaphthalene-enrichment, was the only strain belonging to Alphaproteobacteria.

A greater variety of microbes was isolated from the UV-treated control mesocosm (BIV) on-board the ship; in addition to *Pseudoalteromonas*, a member of the genus *Pseudomonas* (90% identity to *Pseudomonas stutzeri* 3C68, 93.2% identity to *Pseudomonas* sp. W1-1) and several isolates belonging to the genus *Halomonas* (99.4–99.8% identity) were isolated from 1-methylnaphthalene enrichments from mesocosm BIV.

### Utilization of different growth substrates

A growth experiment was set up in order to investigate the range of carbon sources that these isolates could utilize for growth, mainly focusing on hydrocarbons. Based on the phylogenetic tree (Fig. 3), eight phylogenetically distinct *Pseudoalteromonas* strains (two from each hydrocarbon enrichment), six phylogenetically distinct strains of *Halomonas*, and the *Glaciecola* and *Roseovarius* strains, were selected.

**Fig 4:**
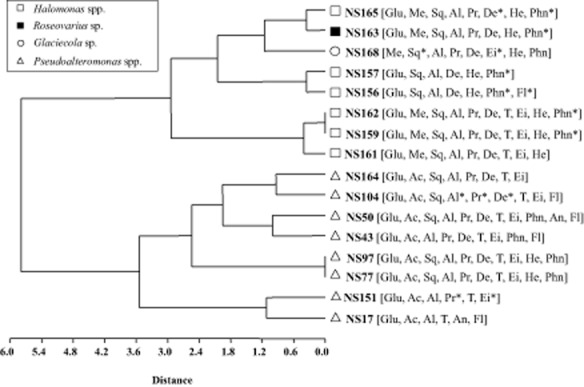
Phenogram of sequenced isolates based on the pattern of growth substrate utilization. All negative for Biphenyl, Pyrene, Toluene, Benzene, Cyclohexane and Sodium Benzoate. All positive for Pyruvate, L-Alanine, L-Arginine, Forties crude oil. Square brackets contain substrates utilized by corresponding isolate. (Glu = Glucose; Al = L-Alanine; Ac = K-acetate; Me = Methanol; Sq = Squalane; T = Tetradecane; Pr = Pristane; De = Decane; He = Hexacosane; Phn = Phenanthrene; Ei = Eicosane; Fl = Fluorene; An = Anthracene). * = One out of two cultures grew on the substrate.

The results of these assays are summarized in Fig. 4. All the isolates were able to grow with Forties crude oil, pyruvate and the amino acids alanine and arginine as sole carbon sources. With the exception of strain *Glaciecola* NS168 all strains grew on glucose. Acetate and tetradecane supported growth of all *Pseudoalteromonas* strains, while only three strains of *Halomonas* (NS159, NS161, NS162) grew with tetradecane. However, no isolates grew on benzoate, cyclohexane, benzene, toluene, pyrene or biphenyl; while methanol supported growth of four strains of *Halomonas* (NS159, NS161, NS162 and NS165), *Glaciecola* (NS168) and *Roseovarius* (NS163). Some isolates grew on a wide range of hydrocarbons whereas others seemed to specialize in particular classes of hydrocarbons. For example, *Pseudoalteromonas* strain NS50 grew on linear alkanes (decane, tetradecane and eicosane), branched alkanes (pristane and squalane) and PAHs (phenanthrene, anthracene and fluorene). *Pseudoalteromonas* strain NS151, *Halomonas* strains NS159, NS161, NS162 and *Pseudoalteromonas* NS164, isolated from 1-methylnaphthalene enrichments, did not grow on any other aromatic hydrocarbon but grew on *n*-alkanes and branched alkanes. Generally, linear alkanes supported the growth of most of the isolates.

### Crude-oil biodegradation by selected isolates

The components of crude oil consumed by isolates NS163 (99% 16S rRNA gene sequence identity to *Roseovarius* sp. AP6), NS168 (100% identity to *Glaciecola* sp. MOLA 37), NS159 (99.6% identity to *Halomonas* sp. MAN K33) and isolate NS164 (99% identity to *Pseudoalteromonas elyakovii*) were identified by growing them on 1% v/v weathered Forties crude oil for 12 days (except strain NS164 that was incubated for 28 days) and measuring the hydrocarbon components remaining. There was a significant reduction of Total Petroleum Hydrocarbon (TPH) (Tukey's HSD: *P* < 0.05) in all cultures, confirming that all the isolates were oil degraders, with isolates NS159 (*Halomonas* sp.) and NS168 (*Glaciecola* sp.) exhibiting similar degradative potentials (data not shown).

These four isolates exhibited a preference for alkanes to PAHs. Total alkanes significantly reduced by 36.9, 42.1, 44.7% for strains NS163, NS168 and NS159 respectively after 12 days of incubation, and by 54% for strain NS164 after 28 days (Tukey's HSD: *P* < 0.05). In contrast, total PAH values were unchanged.

**Fig 5:**
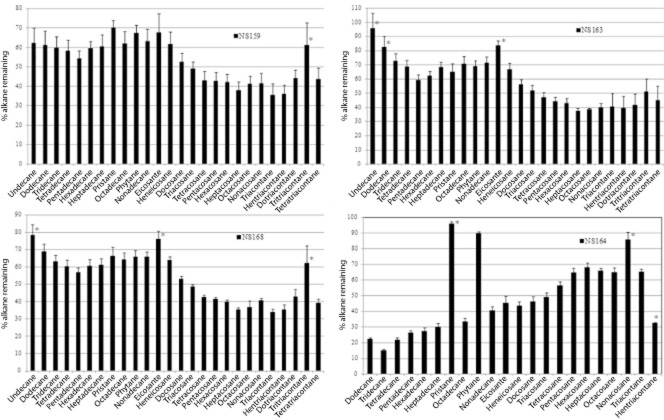
Changes in alkane concentration in Forties crude oil after cultivation with four different isolates. The values represent percentage remaining of each alkane component relative to the negative control after 12 days of incubation (28 days for strain NS164). Vertical bars show the standard error; *n* = 3. The asterisk denotes no significant degradation (Tukey's HSD: *P* > 0.05; Dunnett's two sided: *P* > 0.05). The four strains are: NS159 (99.6% 16S rRNA sequence identity to *Halomonas* sp. MAN K33), NS163 (99% 16S rRNA sequence identity to *Roseovarius* sp. AP6), NS168 (100% 16S rRNA sequence identity to *Glaciecola* sp. MOLA 37) and NS164 (99% 16S rRNA sequence identity to *Pseudoalteromonas elyakovii*).

The extent of biodegradation of individual alkane components (C_11_–C_33_) in Forties crude oil by the four isolates was assessed (Fig. 5). Strain NS159 significantly degraded all alkane components although those in the range of C_18_–C_20_ (including the branched-chain alkanes) were least degraded. Strain NS163 and NS168 did not significantly degrade C_11_, C_12_ and C_20_ *n*-alkanes (Tukey's HSD: *P* > 0.05), but all other alkanes were degraded. *Pseudoalteromonas* strain NS164 had a different degradation profile, significantly degrading most of the alkane components (Tukey's HSD: *P* < 0.05) except pristane and C_29_ and C_31_ alkanes.

## Discussion

### Open-sea experimental spill versus the on-board mesocosms

We took this rare opportunity to investigate the effects of a light crude-oil spill on the bacterial community in the marine water column under natural conditions. When sampling from inside the experimental spill, it was essential to minimize disturbance of the oil so that other measurements would not be compromised and to avoid breakup of the slick, and so sampling was performed 3 m below the surface. The open-sea oil slick had dissipated by 32 h, and, for the reasons described by Brussaard and colleagues (2010), production of a larger, contained slick was not feasible. Therefore, in parallel, on-board mesocosms were established to simulate a more severe oil spill in a restricted area, e.g. in a harbour or bay. DGGE analysis revealed that bacterial communities in the on-board mesocosms changed rapidly upon oil addition, with a decline in most SAR11 phylotypes and a concomitant increase in *Pseudoalteromonas* spp. (see later discussion). In contrast, the bacterial community composition in the water column inside and outside the experimental oil spill did not change over the course of the 32-hour experiment. Three factors are likely to have contributed to this difference between open-sea and enclosed-mesocosm experiments. 1) The timescale of the on-board mesocosm was much longer (7 days), and indeed most differences became apparent after 2 days, and so insufficient time had elapsed to allow a large population of hydrocarbon degraders to develop in the open-sea experiment. 2) The availability of oil was lower in the open-sea experiment, due to: horizontal spreading of the Arabian light crude oil, lower oil loading per unit area, and greater depth of sampled water, and thus distance from the oil source. 3) In contrast to the simulation of an enclosed spill, the open sea would have received a flow of seawater from uncontaminated areas, containing a new supply of nutrients and microbes. These findings emphasize that complementary approaches, including experimental oil spills at sea, are required to investigate the microbial responses to crude oil; and that scaling-up data from mesocosms to the open sea requires careful consideration.

### Negative impact of crude oil on the SAR11 clade

Dominant bands in the uncontaminated mesocosm (BI) and in the less-oiled mesocosm (BII) or at early stages in the most oiled one (BIII) were similar (97–100%) to SAR11, a clade of Alphaproteobacteria and the most abundant phylotype in the surface ocean (Morris *et al*., 2002). SAR11 varies widely in spatial and temporal abundance, but generally represents the dominant bacterioplankton taxon in the North Sea, constituting up to 37% (Giebel *et al*., 2011) or 47% (Sperling *et al*., 2012) of total bacterial 16S rRNA genes.

*Candidatus* Pelagibacter ubique strain HTCC1062 is the first cultured member of the clade and has one of the smallest genomes of autonomously replicating bacteria (Giovannoni *et al*., 2005). Evidence from this strain and *in-situ* studies have shown that SAR11 bacteria are predominantly chemoorganoheterotrophs, utilizing glucose, amino acids, trimethylamine *N*-oxide and dimethylsulfoniopropionate (Malmstrom *et al*., 2004; Laghdass *et al*., 2012; Lidbury *et al*., 2014), but when starved of organic carbon, growth is enhanced by light-energy capture via proteorhodopsin (Steindler *et al*., 2011). Importantly, our study has provided experimental evidence that a prolonged dose of light crude oil has a negative impact on this bacterial clade that has such a pivotal role in marine ecology and biogeochemical processes: most SAR11-derived bands decreased in intensity with time in the oiled mesocosms compared with the non-oiled mesocosm, and were absent after day 4 in the mesocosm receiving most oil (BIII). The detrimental effects of oil on SAR11 phylotypes were also observed in diesel-polluted mesocosms containing surface water from a coastal area of southwest Mallorca (Lanfranconi *et al*., 2010). Nogales and colleagues (2007) also noted the absence of SAR11 in polluted harbour waters. Moreover, Redmond and Valentine (2011) found SAR11 to be among the dominant bacteria in surface water collected from three sites of the Deepwater Horizon spill that had a thin oil sheen, but they were undetected or represented by < 1% of 16S rRNA sequences in two sites with a thicker oil coating. Also in the Gulf of Mexico, but this time in the oil plume in deeper water, most of the abundant taxa showed an increase in gene expression; in contrast SAR11 transcript abundance was lower inside than outside the plume, but not significantly so (Rivers *et al*., 2013). Little attention is given to the impact of crude oil on marine bacteria, yet we showed that there are clear dose- and time-dependent effects on SAR11. It will be important to determine what effect spills of different magnitude may have on such microbes, as well as the resulting impact on cycling of elements (e.g. N, P and Fe) that are required for efficient hydrocarbon degradation. The mechanisms by which crude oil inhibits SAR11 cells remain to be elucidated, but may include: direct interaction of hydrocarbons with cells, e.g. damaging membranes; reduced light availability, potentially affecting sensory processes or energy generation; and/or indirect processes, such as enhanced competition with hydrocarbon-degrading microbes for nutrients.

### The importance of nutritionally diverse bacteria, such as Pseudoalteromonas spp., in hydrocarbon degradation in the marine water column

*Pseudoalteromons* spp. are ubiquitous in marine environments, representing on average 2.6% of the surface bacterioplankton along a latitudinal gradient (Wietz *et al*., 2010), but less than 1% in one location in the southern North Sea (Eilers *et al*., 2000). *Pseudoalteromonas*-related bands became dominant in oiled mesocosms (BII, BIII, BIV), and *Pseudoalteromonas* spp. were the most commonly isolated bacteria, including from the open-sea spill. This, coupled with the demonstrated ability of these isolates to degrade a wide range of hydrocarbons, implies that they were the main microbes responsible for hydrocarbon degradation in the water column. No obligate hydrocarbonoclastic bacteria (OHCBs) were detected or isolated from the experimental spill or the mesocosms. This was not due to the choice of media, because in another study of mudflat sediments using the same procedures, OHCBs belonging to the genera *Alcanivorax, Cycloclasticus* and *Thalassolituus* were isolated in abundance (Sanni *et al*., unpublished). OHCBs such as *Alcanivorax borkumensis* and *Marinobacter hydrocarbonoclasticus* form biofilms that aid their attachment to oil-water interfaces where they carry out degradation (Schneiker *et al*., 2006; Duran, 2010), and so they may well have been present on the unsampled oil-water surface. However, an intriguing possibility is that the anti-biofilm activity of *Pseudoalteromonas* (Dheilly *et al*., 2010; Klein *et al*., 2011) may have inhibited oil-attached OHCBs.

The closest known relatives of the oil-degrading *Pseudoalteromonas* strains isolated in this study (e.g. *P. haloplanktis*, *P. elyakovii*, *P. distincta*, *P. atlantica*) are not known to degrade hydrocarbons. However, representatives of this genus have been detected in a variety of oil-contaminated environments and oiled microcosms, and shown to degrade hydrocarbons. Notably, in one of the two surface water samples from the Gulf of Mexico that had a thick layer of oil, and that was mentioned previously because of the absence of SAR11 (Redmond and Valentine, 2011), *Pseudoalteromonas* constituted 93% of the 16S rRNA sequences, and strains were isolated from plume water samples during the Deepwater Horizon spill (Gutierrez *et al*., 2013). *Pseudoalteromonas* spp. increased in abundance in deeper waters of the Gulf-of-Mexico plume, after hydrocarbon exposure, especially after alkanes had been depleted and aromatics remained (Dubinsky *et al*., 2013). Also, *Pseudoalteromonas* clones were detected in seawater microcosms amended with crude oil and incubated at 4°C and 20°C (Yakimov *et al*., 2004b; Coulon *et al*., 2007), and in PAH enrichment-microcosms of seawater amended with nutrients over a period of 9 weeks (McKew *et al*., 2007a). *Pseudoalteromonas* spp. have been isolated from phenanthrene, chrysene and naphthalene-enrichment cultures of marine sediments (Melcher *et al*., 2002; Hedlund and Staley, 2006; Cui *et al*., 2008). The biodegradation potential of *Pseudoalteromonas* spp. for both aliphatic and aromatic compounds of crude oil has been confirmed in a consortium deriving from arctic ice and seawater (Deppe *et al*., 2005) and in a consortium from the Korean Western coast (Cho and Oh, 2012), whereas a strain of *Pseudoalteromonas* has been found to preferentially metabolize short-chain alkanes in the range of C_12_–C_20_ in arctic sediments (Lin *et al*., 2009). All of our *Pseudoalteromonas* strains grew on a range of carbon and energy sources, and varied in the breadth of utilization of hydrocarbons, with all but three strains able to grow on straight-chain and branched alkanes as well as PAHs, but none grew on toluene. In contrast, OHCBs, like *Alcanivorax* and *Cyloclasticus* spp., tend to be more specialized, degrading alkanes and PAHs, respectively (see Cappello and Yakimov, 2010; Staley, 2010).

Interactions between microbes are as important in oil degradation as they are in other biogeochemical processes (McGenity *et al*., 2012), and the ability of *Pseudoalteromonas* spp. to remove potential competitors has been mentioned previously. *Pseudoalteromonas* spp. may also be beneficial to co-existing microbes by producing EPS that has the potential to serve as a biosurfactant (Nichols *et al*., 2005; Saravanan and Jayachandran, 2008; Cho and Oh, 2012). It will be important to elucidate the relative contribution of generalists like *Pseudoalteromonas* and specialists like *Alcanivorax*, and to understand to what extent they interact. *Pseudoalteromonas* spp. will be at an advantage owing to their higher abundance in unpolluted seawater, allowing them to become established more rapidly after an oil spill; however, the burden of carrying genes that are not required for hydrocarbon biodegradation is likely to make them less competitive over time.

### Other hydrocarbon-degrading isolates

Whereas *Pseudoalteromonas* was both shown to be abundant in oiled mesocosms and readily cultivable on hydrocarbon-enriched marine media, hydrocarbon-degrading strains from other genera were also isolated but not shown to have increased in abundance in the oiled mesocosms. Six strains were related to species of *Halomonas* (more than 99% percent identity), all of which derived from UV-treated and oiled mesocosm BIV and had been enriched with 1-methylnaphthalene as sole carbon source. Like *Pseudoalteromonas* strains, they grew on a wide range of hydrocarbons, including branched and straight-chain alkanes and PAHs. GC-MS analysis of one strain demonstrated metabolism of almost all the alkane components of crude oil but not the aromatic components within 12 days, implying a preference for alkanes when both alkane and PAHs are present as crude oil components. *Halomonas* spp. have been reported to degrade aromatic (Melcher *et al*., 2002; Garcia *et al*., 2004; Cui *et al*., 2008) and aliphatic hydrocarbons (Pepi *et al*., 2005; Mnif *et al*., 2009) in marine and hypersaline environments.

One strain had 99% 16S rRNA sequence identity with two members of the Roseobacter clade: *Roseovarius* sp. AP6 which was isolated by Alonso-Gutierrez and colleagues (2009) from shoreline samples contaminated with oil from the *Prestige* spill, and *Roseovarius* sp. DG944 found in association with an alga, *Gymnodinium catenatum*, which causes paralytic shellfish poisoning in marine waters (Green *et al*., 2004). *Roseobacter* spp. were found in abundance in both *n-*alkane- (McKew *et al*., 2007a) and oil- (Brakstad and Lødeng, 2005) enrichments of seawater and also in oil-contaminated seawater (Prabagaran *et al*., 2007). Similarly, species of *Roseovarius* have been implicated in the degradation of PAHs (Harwati *et al*., 2007; Wang *et al*., 2008; Vila *et al*., 2010), *n-*alkanes and pristane (Harwati *et al*., 2007).

Species of *Glaciecola* are usually found in polar seas and their coastal environments, Arctic and Antarctic sea-ice in association with marine invertebrates (Romanenko *et al*., 2003; Yong *et al*., 2007) and clones have been detected in oil-contaminated samples from such environments (Prabagaran *et al*., 2007; Brakstad *et al*., 2008; Røberg *et al*., 2011). Yakimov and colleagues (2004b) also isolated a strain of *Glaciecola* from tetradecane-enrichments of Antarctic seawater. However, these studies did not present evidence to support the direct participation of these species in hydrocarbon degradation, whereas our *Glaciecola* strain NS168 grew on most of the *n*-alkanes tested and on pristane.

It is noteworthy that the four isolates (*Roseovarius* sp. strain NS163, *Glaciecola* sp. strain NS168, *Halomonas* sp. strain NS159 and *Pseudoalteromonas* sp. strain NS164) did not degrade any of the PAH components of crude oil during the period of incubation (as evidenced by GC-MS analysis), even though they were all isolated on 1-methylnaphthalene-enrichments and the first three strains grew on phenanthrene in the growth substrate assay. This implies that the strains have a preference for alkanes when both alkane and PAHs are present as crude oil components but could metabolize some PAHs in the absence of alkanes.

### Selection of *Alteromonas* sp. by UV-light treatment

Although the primary purpose of the UV-treated mesocosm (BIV) was to allow analysis of *in-situ* hydrocarbon biodegradation, it was a source of several strains of hydrocarbon-degrading microbes. Hydrocarbons were a selective force in this mesocosm, but also a dense band indicating the presence of *Alteromonas*, not detected in other oiled mesocosms, could be a consequence of the resistance of representatives of this genus to ultraviolet light (Agogué *et al*., 2005) and their ability to grow rapidly on the organic matter released from cells killed by UV light in the same way that they are often associated with phytoplankton blooms (Tada *et al*., 2012) and exudates (Nelson and Carlson, 2012).

### Environmental implications

Several factors have been proposed to explain the patchy horizontal distribution of important marine taxa, such as SAR11, in marine environments (Sperling *et al*., 2012); however our study shows that consideration should be given to oil-pollution incidents. Also, a ten-fold enrichment of *Pseudoalteromonas* spp., and absence of SAR11, in the sea-surface microlayer compared with underlying North Sea water (Franklin *et al*., 2005), may in part be explained by the accumulation of hydrocarbons in the sea-surface microlayer (Wurl and Obbard, 2004) and the demonstrated sensitivity of SAR11 to, and predilection of *Pseudoalteromonas* spp. for, hydrocarbons.

It has been proposed that ecological damage caused by oil spills might be reduced by the addition of microbes like *Alcanivorax* spp. which, although naturally present in contaminated sites, require considerable time to reach population densities needed for maximum biodegradation rates. The work presented here shows that such bioaugmentations may only address biofilm-biodegradation of oil-slick or oil-droplet contamination and not dispersed hydrocarbons in the water column. Thus, bioaugmentation strategies may require multiple species with different specialities, including oil-attached specialists like *Alcanivorax* and planktonic generalists such as *Pseudoalteromonas*.

## Experimental procedures

### Sample site and sample collection

#### The experimental oil spill

Details of the permitted, controlled, experimental spill and on-board mesocosms are provided by Brussaard and colleagues (2010). As part of an EC project FACEiT (Fast-Advanced Cellular and Ecosystem Information Technologies), an experimental oil spill was organized by the Royal Netherlands Institute for Sea Research (NIOZ), Texel, in collaboration with the Netherlands Ministry of Transport, Public Works and Water Management, RWS Noordzee (RWS-NZ); the spill was carried out on 8 May 2008 in Netherlands Exclusive Economic Zone waters. To aid the visualization and tracking of the oil slick, 5 kg of the fluorescent dye rhodamine WT (25 L 20% w/v) was mixed with 1 m^3^ of seawater and discharged together with 5000 L of Arabian Light crude oil into Dutch waters. In addition to the dye, two drifting buoys were used to locate the position of the oil slick especially when the rhodamine had diluted beyond the limit of detection and it thus became difficult to follow the slick. Time zero (0 h) corresponds with the end-point of oil and dye addition, which took approximately 30 min. In order to prevent RV Pelagia from disturbing the experimental spill, a rubber boat was dispatched to collect water samples from inside the spill (Spill-in) using a specially designed sampler that gently pumped seawater from 3 m depth into a metallic jerry can. The tube used was attached to a metallic inlet that was passed closed through the surface oil layer, preventing oil droplets from contaminating the sample. Samples were taken at 0, 3, 9, 12, 24 and 32 h after addition of oil. Within 30 min of these time points, corresponding control samples were collected from waters at least 500 m from the spill with no apparent oil on the surface, and ensuring no cross-contamination with the added oil by accounting for wind, tide and current. These samples, called ‘Spill-out’, were taken at a depth of 1.5 m using Niskin bottles (10 L each) mounted on a Rosette sampler equipped with Seabird conductivity-temperature-depth (CTD) sensors and a natural chlorophyll autofluorescence sensor.

#### Accidental slicks

Samples were collected from oil slicks encountered in shipping lanes within a section of the Dutch EEZ rich of oil platforms (southeast of Dogger bank) (Acc-in) at 1.5 m water depth. Corresponding control samples (Acc-out) were collected in clean waters outside the slicks at the same depth.

#### Oil-enriched enclosed mesocosms

In addition to the experimental spill, four 1 m^3^ mesocosms (called Cube vessels) were set up on board the RV Pelagia on the 6^th^ May, 2008. The Cube vessels were filled with seawater (1000 L) from the Dogger Bank, a biologically productive location containing waters with no obvious oil contamination and high biomass. Cube 1 (BI) served as the non-oiled control, Cube 2 (BII) contained 2 L of Arabian Light Crude oil, Cube 3 (BIII) contained 5.5 L of the same crude oil, and Cube 4 (BIV) contained 2 L of oil, but was treated with UV light to sterilize the mesocosm (‘killed control’). Each Cube vessel had three taps: at the top, middle and bottom. As the level of water in each mesocosm reduced, samples were taken from the tap closest to the surface of the water (approximately 15–20 cm from the surface).

The mesocosms were sampled on the 7^th^ May, 2008 before the addition of oil at 08:15 (t = 0), then at 2 h, 6 h, 12 h after oil addition. Further samples were obtained daily at 13:00 for the next 7 days.

### Initial processing of samples

Aliquots of seawater sampled from all environments were immediately inoculated into hydrocarbon-enrichment media while the rest were stored on board the RV Pelagia and transported at 4°C back to the University of Essex lab. Microbial cells were also immediately collected on 0.2 μm pore size filters by filtering 1.2 L of seawater samples with Nalgene bottle-top filter units. Seawater samples (pristine, experimental spill and accidental spills) were filtered with Sartorius gridded cellulose nitrate filters (47 mm diameter, 0.2 μm pore size) while samples from the Cube vessels were filtered with Durapore PVDF filters (47 mm diameter, 0.22 μm pore size). The filters were initially stored at 4°C for 12 h in centrifuge tubes (50 ml) containing RNA*later*, and then frozen at − 80°C.

### Hydrocarbon enrichment cultures

Hydrocarbon enrichment cultures in liquid media were established by inoculating water samples (200 μl) into tubes containing 10 ml of ONR7a medium (Dyksterhouse *et al*., 1995) using 1% v/v filter-sterilized tetradecane, weathered Forties crude oil, pristane or 1-methylnaphthalene as sole carbon sources. Tubes containing ONR7a medium with hydrocarbons had been prepared, capped and crimp-sealed with PTFE-lined silicon septa prior to inoculation. Subculturing was done by carrying out 10-fold serial dilutions of the cultures and spreading 50 μl from the 10^−2^, 10^−5^ and 10^−8^ dilutions on ONR7a agar plates containing sterile GF/C filter papers soaked with 125 μl of the hydrocarbons on the lids of the Petri dishes.

All hydrocarbon enrichment cultures using solid media were prepared with washed agar. These enrichments were prepared by spreading 100 μl of seawater samples (that had been preserved at 4°C for a maximum of three weeks) onto ONR7a agar with hydrocarbon-soaked GF/C filter papers placed on the lids of the Petri dishes. Each of the four hydrocarbons (125 μl) was used as sole carbon source. All cultures were incubated at 12°C, representing the *in-situ* temperature at the time of sampling.

### Nucleic acid extraction, polymerase chain reaction, denaturing gradient gel electrophoresis, and sequencing

DNA was extracted from cultures by boiling the bacterial cells in diethyl pyrocarbonate-treated water (DEPC water), followed by PCR amplification of bacterial 16S rRNA genes using primers described by Lane (1991). The 16S rRNA amplicons of bacterial isolates were sent to GATC Biotech (http://www.gatc-biotech.com/en/index.html) for sequencing.

DNA from filtered water was extracted following bacterial cell lysis with phenol : chloroform: isoamylalcohol (25:24:1 v/v) as described by McKew and colleagues (2007a). PCR of bacterial 16S rRNA gene from this DNA was performed using primers described by Muyzer and colleagues (1993), followed by denaturing gradient gel electrophoresis (DGGE) employing a denaturing gradient from 40 to 60% (McKew *et al*., 2007a). Bands were excised and sequenced on an ABI Prism 3100 Genetic Analyser.

Full details of these methods and associated analyses are given in the supporting information.

### Accession numbers

The DNA sequences reported in this study were deposited in the EMBL database under the accession numbers HE961976 to HE962017.

### Growth substrate assays

The ability of selected isolates to utilize different carbon sources was examined by growing the isolates (100 μl) in duplicate tubes containing sterile ONR7a liquid medium (10 ml) and various carbon sources (0.1% v/v). The growth substrates included Forties crude oil, *n*-alkanes (decane, tetradecane, eicosane and hexacosane), branched alkanes (pristane and squalane), cyclic alkane (cyclohexane), aromatic hydrocarbons (benzene, toluene, phenanthrene, anthracene, pyrene, fluorene and biphenyl), amino acids (L-alanine and L-arginine), D-glucose, Na-benzoate, Na-pyruvate, K-acetate and methanol. Details are provided in the supporting information.

### Biodegradation of crude oil by selected isolates

Detailed biodegradation studies were carried out on four selected isolates. Washed, suspended cells (200 μl) were inoculated in triplicate into serum bottles (125 ml) containing sterilized ONR7a medium (20 ml) with weathered Forties crude oil (1% v/v) and incubated on a rotary shaker (Innova 2300; New Brunswick Scientific) in the dark at 100 rpm for 12 days (isolate NS164 was incubated for 28 days of 12 h light-dark cycles). After incubation, the cultures were vigorously vortexed for 10 s to dislodge bacterial cells from the crude oil and centrifuged in a Sorvall Biofuge Stratos centrifuge at 8,500 *g* for 15 min at 4°C to pellet cells. Total hydrocarbon was solvent extracted from the liquid contents of the serum bottles as described by Coulon and colleagues (2007), and hydrocarbons were quantified using a Thermo Trace GC gas chromatograph attached to a Thermo Trace DSQ® mass spectrometer. Details are given in the supporting information.

### Statistical analysis

Similarity between DGGE profiles was calculated based on a manually produced binary matrix of the presence/absence of DGGE bands. A proximity matrix based on the Pearson correlation coefficient was used to construct a Multidimensional Scaling (MDS) plot in XLSTAT Version 2008.1.02. A phenogram based on the growth substrate utilization pattern of sequenced isolates was generated with PAST (Paleontological statistics) 2.17 software package (Hammer *et al*., 2001) using the Ward's method and Euclidean distance to demonstrate the dissimilarities in substrate utilization by the isolates. Significant hydrocarbon degradation was determined by analysis of variance coupled with Tukey's HSD and Dunnett's two sided tests. These tests were performed with XLSTAT 2011 (Addinsoft™).

## Supporting Information

**Fig. S1.** DGGE profiles of bacterial communities in the non-oiled (BI) and the most oiled (BIII) on-board mesocosms over time, based on 16S rRNA gene. **Appendix S1.** Supporting experimental procedures.
