# Supplementary material for: Generalist hydrocarbon-degrading bacterial communities in the oil-polluted water column of the North Sea
Source: Microb Biotechnol. 2014 Sep 24;8(3):434–47. doi: 10.1111/1751-7915.12176 (PMC4408176; doi:10.1111/1751-7915.12176)
Supplement: Supplementary file 1 [file mbt20008-0434-sd1.docx]

**Supporting Information**

**Generalist hydrocarbon-degrading bacterial communities in the oil-polluted water column of the North Sea**

**Chronopoulou, Panagiota-Myrsini^1 * †^, Sanni, Gbemisola O.^1 *^, Silas-Olu, Daniel I.^1^, van der Meer, Jan Roelof^2^, Timmis, Kenneth N.^3^, Brussaard, Corina P.D.^4 ‡^ and McGenity, Terry J.^1^**

1. School of Biological Sciences, University of Essex, Wivenhoe Park, Colchester CO4 3SQ, UK.

2. Department of Fundamental Microbiology, University of Lausanne, 1015 Lausanne, Switzerland.

3. Institute of Microbiology, Technical University Braunschweig, Spielmannstrasse 7, D-38106 Braunschweig, Germany.

4. Department of Biological Oceanography, Royal Netherlands Institute for Sea Research (NIOZ), 1790 AB Den Burg, The Netherlands.

* Equal contribution to the work

For correspondence, e-mail Dr. Terry McGenity at [tjmcgen@essex.ac.uk](mailto:tjmcgen@essex.ac.uk)

Tel.: (+44) 1206 872535

Fax: (+44) 1206 872592

**Running title**

Generalist marine hydrocarbon-degrading bacteria

**^†^** Current address: School of Biological and Chemical Sciences, Queen Mary University of London, Mile End Road, London, E1 4NS, UK.

^‡^ Current address: IBED, University of Amsterdam, 1090 GE, Amsterdam. The Netherlands.

**Experimental Procedures**

***Chemicals***

Reagents such as D-glucose, sodium benzoate, potassium acetate, methanol and L-arginine were purchased from Fisher. Sodium pyruvate, squalane, pristane, cyclohexane, decane, anthracene, phenanthrene, fluorene and biphenyl were obtained from Sigma-Aldrich. L-alanine, tetradecane, eicosane, hexacosane, benzene, pyrene and toluene came from Acros Organics. Deuterated alkanes and PAHs were purchased from Sigma-Aldrich. All reagents were analytical grade.

***Nucleic acid extraction from cultures and filtered water***

After a series of subculturing steps (between 2 and 6 steps), nucleic acids were extracted from single colonies by boiling for 30 min in sterile diethyl pyrocarbonate-treated water (DEPC water) to lyse the cells. For those strains whose cells were not lysed by boiling, total nucleic acid was extracted as described by McKew *et al.* (2007). The nucleic acid pellets were washed twice with 200 µl of 70% v/v ethanol, allowed to air dry, re-suspended in sterile DEPC-treated water (0.1% v/v DEPC in reverse osmosis water, left for 6 h or overnight at room temperature, then autoclaved) and stored at -20°C.

For the extraction of nucleic acids from filtered water filters were taken out of the RNA*later* solution and were cut in two halves using a sterile scalpel. One half of the filter was used for the extraction and the other half was kept in RNA*later* and stored at -80°C.Nucleic acids were extracted using a phenol:chloroform:isoamylalcohol method (25:24:1 v/v) as described by McKew *et al.* (2007).

***Polymerase Chain Reaction***

Bacterial 16S rRNA genes deriving from cultures were amplified using 16S 27F/1492R primer pair (27F: 5′-AGAGTTTGATCMTGGCTCAG-3′; 1492R: 5′-

TACGGYTACCTTGTTACGACTT-3′) (Lane, 1991). PCR was carried out in a Gene Amp® PCR system 9700 thermocycler (Applied Biosystems). A 50 µl PCR reaction master mix contained 1 mM dNTP mix (Fermentas), 0.4 µM of each primer (Invitrogen), 2.5 units of Taq DNA polymerase, 5 µl of 10× PCR buffer, 10 µl of Q solution (Qiagen) and 1 to 4 µl of template DNA. PCR cycling conditions were 94°C for 2 min, and 30 cycles of 94°C for 1 min, 55°C for 1 min and 72°C for 2 min. A final elongation step at 72°C for 10 min was performed. Amplification of the 16S rRNA gene was confirmed by agarose gel electrophoresis (1% w/v in 1 × TAE buffer at 100 V) and ethidium bromide staining.

Amplification of the nucleic acids extracted from filtered water was performed using primers described by Muyzer *et al*. (1993) and PCR conditions described by McKew *et al.* (2007).

***Denaturing gradient gel electrophoresis analysis***

Denaturing gradient gel electrophoresis (DGGE) of amplified bacterial 16S rRNA genes from filtered water was performed with Bio-Rad D Code system employing a denaturing gradient from 40 to 60% (McKew *et al.*, 2007), followed by band excision and re-amplification as described by Coulon *et al.* (2012). The re-amplified PCR products were purified using the QIAquick PCR Purification Kit (Qiagen) according to the manufacturer’s protocol. Sequencing was performed on an ABI Prism 3100 Genetic Analyser, using the BigDye® Terminator v3.1 cycle sequencing kit (Applied Biosystems) in accordance with the manufacturer’s protocol. Sequences were checked using the Chromas software (Technelysium Pty Ltd) and contigs were assembled in the program BioEdit (version 7.0.0). Sequences were compared to the NCBI database using the Basic Local Alignment Search Tool (BLAST).

***Sequencing of isolates***

PCR products obtained from the 16S rRNA genes of the bacterial isolates were purified with the QIAquick PCR purification kit (Qiagen) following the manufacturer’s protocol, and the amplicons were sent to GATC Biotech (http://www.gatc-biotech.com/en/index.html) for sequencing using the forward primer 16S 27F. The resulting partial 16S rRNA sequences of the isolates were compared against the EMBL Nucleotide Sequence Database for the identification of closest relatives using the FastA programme. Sequences were aligned using the ClustalW application (Thompson *et al*., 1994) of the Bioedit program. Phylogenetic analysis was performed with the PHYLIP package (Felsenstein, 2004), using the Jukes and Cantor model of nucleotide substitution, the neighbour-joining algorithm for tree construction, and bootstrap resampling of 1000 data sets.

***Growth substrate assay***

The isolates were initially grown on ONR7a agar plates containing 0.1% Na acetate and 0.01% yeast extract as carbon source (0.1% yeast extract and 0.5% tryptone was added for the growth of the *Pseudoalteromonas*-like isolates), and, after purity was ascertained by checking under the stereomicroscope, colonies were transferred to 1.5-ml microfuge tubes with 1 ml of ONR7a liquid medium. The cells were washed twice in ONR7a liquid medium by centrifuging at 2,800 *g* to remove traces of carbon source and finally resuspended in 1 ml of ONR7a medium.

Autoclaved stock solutions (100 g L^-1^) of D-glucose, Na-benzoate, Na-pyruvate, K-acetate, L-alanine and L-arginine were prepared, and 100 µl of the solutions was added to the tubes containing 10 ml of ONR7a medium. Weathered Forties crude oil, methanol, squalane, pristane, decane, tetradecane, benzene, toluene, cyclohexane and methanol were filter-sterilised through 0.2 µm PTFE syringe filters before adding 10 µl to the liquid media.

Hexacosane (5 mg) and 10 mg of aromatic hydrocarbon (phenanthrene, anthracene, pyrene, fluorene and biphenyl) were first dissolved in 2 ml and 1 ml of acetone, respectively, filter-sterilised and dispensed into empty test tubes. The acetone was allowed to evaporate leaving crystals of the hydrocarbons prior to the addition of ONR7a media (10 ml). Eicosane was added directly to the tubes. All tubes were capped and crimp-sealed with PTFE-lined silicon septa.

The cultures were incubated in the dark at 100 rpm on a rotary shaker (Innova 2300; New Brunswick Scientific) at 20°C. Negative controls with no bacterial inoculum were also prepared and incubated together with the cultures. The tubes were visually inspected daily and for 4 weeks (10 weeks for the aromatic hydrocarbon assay) for signs of growth, based on turbidity.

***Hydrocarbon extraction and GC-MS Analysis***

The capacity to degrade hydrocarbons within Forties crude oil was tested on four isolates. Washed, suspended cells (200 µl) were inoculated in triplicate into serum bottles (125 ml) containing sterilised ONR7a medium (20 ml) with weathered Forties crude oil (1% v/v) and incubated on a rotary shaker (Innova 2300; New Brunswick Scientific) in the dark at 100 rpm. After incubation, the cultures were vigorously vortexed for 10 s to dislodge bacterial cells from the crude oil and centrifuged in a Sorvall Biofuge Stratos centrifuge at 8,500 *g* for 15 min at 4°C to pellet cells

Total hydrocarbon was solvent extracted from the liquid contents of the serum bottles as described by Coulon *et al*. (2007) and the extracts were eluted with a mixture of hexane: dichloromethane (1:1). Deuterated alkanes (C_10_^d22^, C_19_^d40^ and C_30_^d62^) and deuterated PAHs (naphthalene ^d8^, anthracene ^d10^, chrysene ^d12^ and perylene ^d12^) (0.5 µg ml^-1^ and 0.4 µg ml^-1^ respectively) were added to the extracts to act as internal standards. Total hydrocarbons in the extracts were separated, identified and quantified using a Thermo Trace GC gas chromatograph attached to a Thermo Trace DSQ^®^ mass spectrometer. The gas chromatograph was fitted with a splitless injector and a fused silica capillary column (30 × 0.25 mm internal diameter) coated with RTX^®^-5MS (0.25 µm film thickness; Restek Corporation, UK). Helium was used as the carrier gas and 1 μl of sample was injected into the GC. The temperature of the oven was initially set at 85°C for 2 min, then increased to 310°C at the rate of 20°C min^-1^ and then held at this temperature for 30 min. For comprehensive analysis of all hydrocarbons present in the samples, the mass spectrometer was set at full scan mode (m/z range of 50-650); various peaks were quantified by integrating the peaks at specific m/z.

Calibration curves were generated from the quantification of alkanes (0.005 to 0.5 ng μl^-1^) and PAHs (0.010 to 1.0 ng μl^-1^) internal standards and the peaks were identified by comparing their retention times to those of the standards.

**Supplementary Figures**


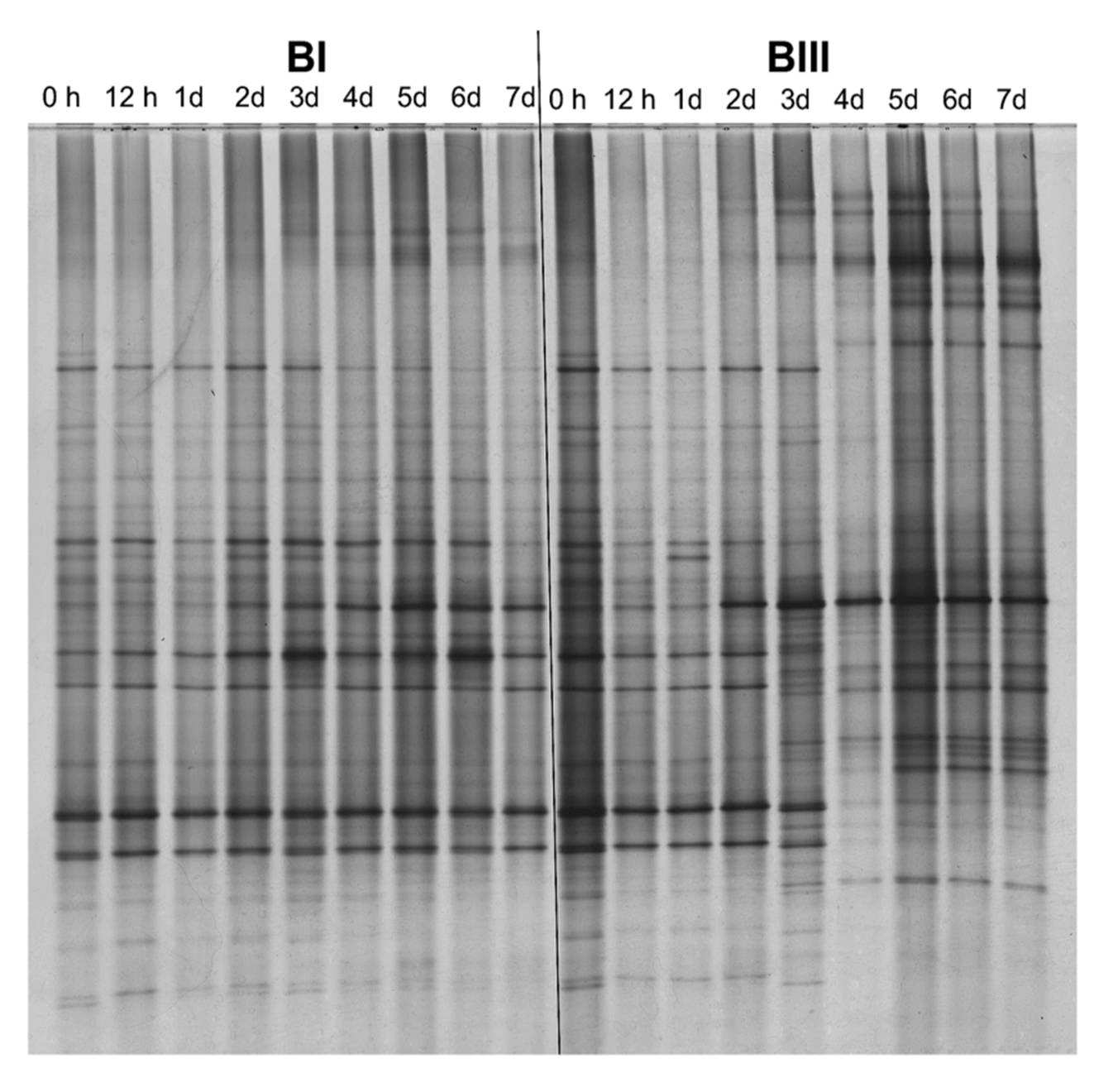


**SI. Figure 1.** DGGE profiles of bacterial communities in the non-oiled (BI) and the most oiled (BIII) on-board mesocosms over time, based on 16S rRNA gene.

**References**

Coulon, F., McKew, B.A., Osborn, M.A., McGenity, T.J., and Timmis, K.N. (2007) Effects of temperature and biostimulation on oil-degrading microbial communities in temperate estuarine waters. *Environ Microbiol* **9**: 177-186.

Coulon, F., Chronopoulou, P., Fahy, A., Païssé, S., Goñi-Urriza, M., Peperzak, L., et al. (2012) Central role of dynamic tidal biofilms dominated by aerobic hydrocarbonoclastic bacteria and diatoms in the biodegradation of hydrocarbons in coastal mudflats. *Appl Environ Microbiol* **78**: 3638-3648.

Felsenstein, J. (2004) *PHYLIP (Phylogeny InferencePackage) Version 3.6*. Distributed by the author. Seattle, WA, USA: Department of Genome Sciences, University of Washington.

Lane, D.J. (1991) 16S/23S rRNA sequencing. In *Nucleic Acid Techniques In Bacterial Systematics*. Stackebrandt, E., and Goodfellow, M. (eds). Chichester: John Wiley & Sons, pp. 115-175.

McKew, B.A., Coulon, F., Osborn, A.M., Timmis, K.N., and McGenity, T.J. (2007) Determining the identity and roles of oil-metabolizing marine bacteria from the Thames estuary, UK. *Environ Microbiol* **9**: 165-176.

Muyzer, G., De Waal, E.C., and Uitterlinden, A.G. (1993) Profiling of complex microbial populations by denaturing gradient gel electrophoresis analysis of polymerase chain reaction-amplified genes coding for 16S rRNA. *Appl Environ Microbiol* **59**: 695-700.

Thompson, J.D., Higgins, D.G., and Gibson, T.J. (1994). CLUSTAL W: improving the sensitivity of progressive multiple sequence alignment through sequence weighting, position specific gap penalties and weight matrix choice. *Nucleic Acids Res* **22**: 4673-4680.
